# The acceptability, adoption and feasibility of mobile health interventions for diabetes and hypertension care among Ghanaian healthcare workers

**DOI:** 10.1016/j.pecinn.2026.100456

**Published:** 2026-01-22

**Authors:** Pearl Aovare, Erik Beune, Felix P. Chilunga, Nicolas Moens, Eric P. Moll van Charante, Charles Agyemang

**Affiliations:** a Department of Public & Occupational Health, Amsterdam UMC, Amsterdam Public Health Research Institute, University of Amsterdam, Meibergdreef 9, 1105 AZ Amsterdam, the Netherlands; b Department of Population, Family and Reproductive Health, School of Public Health, University of Ghana, Legon, Accra, Ghana; c VU University Athena-Institute, Economics, eHealth, and Digital transformation, the Netherlands; d Department of General Practice, Amsterdam UMC, University of Amsterdam, Amsterdam, the Netherlands; e Department of Medicine, Johns Hopkins University School of Medicine, Baltimore, MD, USA

**Keywords:** Mobile health (mHealth), Diabetes care, Hypertension management, Healthcare workers, Technology adoption, Low-resource settings

## Abstract

**Objectives:**

The study explored healthcare workers' experiences using the AfyaPro Connected Care app and identified key enablers and barriers to its adoption for diabetes and hypertension care in Ghana. The study applied the Technology Acceptance Model (TAM) to examine how perceptions of usefulness and ease of use of the app influence adoption and to address the gap in evidence on mHealth uptake by frontline providers in low- and middle-income country (LMIC) health systems.

**Methods:**

A qualitative study was conducted with 20 healthcare workers from two healthcare facilities. Semi-structured interviews, guided by the TAM, explored perceptions of the app's usefulness, ease of use, and behavioral intention. The framework was appropriate for examining individual and contextual drivers of technology adoption in resource-constrained healthcare settings. Interviews were transcribed verbatim and analysed thematically.

**Results:**

Participants reported positive experiences with the app, noting reduced administrative burden, workflow integration, stronger patient-provider interaction and improved continuity of follow-up. The app enhanced access to specialist care, supported self-monitoring of blood pressure and glucose, and boosted confidence through its intuitive design and structured training. However, challenges persisted, including unstable power and internet connectivity, increased data entry workload, limited patient access and digital literacy, and restricted roles for junior staff. Participants recommended clearer roles, regular supervision, refresher training, and decision-support tools to improve sustainability and equitable adoption.

**Innovation:**

This study adopts a user-centered and context-sensitive approach based on provider experiences. It shows how mHealth tools can be fitted into Ghana's healthcare system, where challenges like limited infrastructure and digital literacy affect use. Innovation is seen as adapting tools and systems through digital literacy training, decision-support in provider workflows, and blended care models, helping to build a fairer and more sustainable health system.

**Conclusions:**

Healthcare workers found the mHealth app feasible and acceptable. The findings highlight the potential of digital tools to improve chronic disease care in resource-limited settings. The study demonstrates how contextual factors in LMIC settings reshape key TAM constructs, with clear implications for mHealth policy, scale-up strategies, and refinement of technology adoption theory.

## Highlights

• Healthcare workers support Afyapro app for improved patient outcomes.
• User-friendly app enhances health management and patient empowerment.
• Challenges include connectivity, tech issues for elderly and systemic barriers.
• Recommends decision-support tools, infrastructure fixes, and targeted training.

## 1 Introduction

Ghana and other sub-Saharan African (SSA) nations face a double burden of communicable and non-communicable illnesses [[1], [2], [3]]. Structural challenges, including inadequate healthcare infrastructure, long travel distances, limited funding, and shortages of trained health workers, further undermine access to quality care [2,4].

These challenges are compounded by heavy workloads, weak data management systems, and difficulties in organizing follow-ups and home visits [5] [6]. A recent assessment of primary health facilities in Ghana, for example, showed that while basic equipment and essential medicines for hypertension and diabetes were often available, diagnostic capacity and recent staff training were inadequate [7,8].

Mobile health (mHealth) applications have emerged as promising and affordable solutions to these challenges, particularly for managing chronic diseases such as diabetes and hypertension. Mobile phone–based applications are increasingly viewed as essential tools in modern healthcare, offering functionalities such as patient monitoring, medication reminders, appointment scheduling, and health education. [9] [10].

A recent systematic review of mHealth interventions for diabetes and hypertension in Africa found mixed but generally positive effects, while emphasizing the importance of contextual adaptation and sustained provider engagement [11]. Similarly, qualitative evidence from Kenya, Uganda, and South Africa highlights both the potential of mHealth for chronic disease management and ongoing barriers linked to cost, infrastructure, and training [12,13].

Evidence from high-income countries shows that mHealth tools including messaging, phone consultations, and video conferencing can improve access to care, strengthen interaction between patients and providers, and support disease self-management [10,14].

However, realizing similar benefits in low- and middle-income countries (LMICs) requires addressing significant barriers, including poor connectivity, limited training, costs, and concerns about workflow integration and data security. [15] [16]. Despite growing mHealth adoption in Africa, little is known about how Ghanaian health workers integrate such tools into routine chronic disease care, and how contextual constraints shape adoption through the TAM lens [17]. To address this gap, this study explored the experiences, facilitators, and barriers reported by health workers using an mHealth application to deliver diabetes and hypertension care in Ghana. We specifically selected two healthcare facilities representing contrasting contexts an urban tertiary hospital and a semi-rural district hospital to capture how facility type, patient population, and resource availability influence mHealth adoption and integration into routine workflows.

### 1.1 The objective of the study

Therefore, the main objectives of the current study were to 1) evaluate how health workers relate to and experience mHealth in delivering diabetes and hypertension care services; and 2) identify the facilitating and inhibiting factors related to the implementation and adoption of the mHealth intervention. These objectives are framed around three key implementation constructs: acceptability (how satisfactory and suitable health workers find the intervention), adoption (the extent to which they integrate it into routine practice), and feasibility (the practicality of implementing it, including ease of use and fit with existing workflows).

### 1.2 Overview of the connected care app and pilot implementation

The app (named: Afya Pro Connected Care app) was part of a pilot implementation. The pilot implementation of the app aimed to evaluate its effectiveness as a mobile health intervention for managing chronic diseases, specifically diabetes and hypertension. (https://appadvice.com/game/app/afyapro/1464879369) or https://ecareaccess.org/.

The app incorporates several key functionalities to support both patients and healthcare providers. Monitoring features allow real-time tracking of vital signs, including blood glucose and blood pressure. Communication tools provide a secure channel for patients and providers to exchange messages, support follow-up, and address concerns. Scheduling functionalities help manage appointments and deliver medication reminders, promoting adherence and continuity of care. Additionally, the app offers educational resources to improve patient knowledge and encourage self-management. Grouping these features thematically allows for a clear understanding of how the app addresses different aspects of chronic disease management.

Healthcare workers received structured training to guide patients through the app's functions, demonstrating monitoring, scheduling, and educational tools. Follow-up sessions reinforced training, addressed challenges, and strengthened engagement, ensuring that patients could effectively use the app at home while maintaining open communication with their providers.

The pilot implementation provided valuable insights into the app's usability and its integration into existing health information systems. By collecting data on user interactions and health outcomes, the study identified best practices for scaling the app to a wider population and integrating it into routine workflows. The streamlined main text focuses on the pilot implementation, usability, and thematic app features, while more detailed background, rationale, and technical specifications are provided in Supplementary Appendix 1.

### 1.3 Theoretical framework

**Fig. 1:**
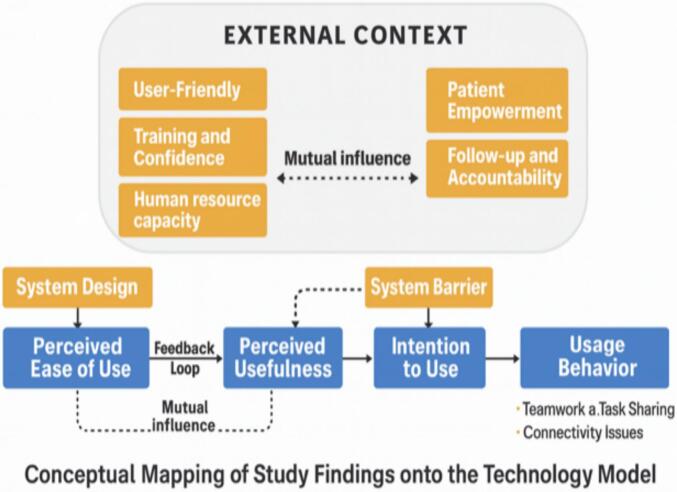
Conceptual diagram showing how study findings map onto TAM constructs; Conceptual diagram illustrating how external contextual factors identified in this study act as modifiers to TAM constructs, shaping perceived ease of use, perceived usefulness, intention to use, and actual usage behaviour. Adapted from the Technology Acceptance Model (Davis, 1989). (Source: Adapted and developed from the Technology Acceptance Model (Davis, 1989).)

This study was guided by Davis's Technology Acceptance Model (TAM), which focuses on perceived ease of use, perceived usefulness, behavioral intention, and actual system use in technology adoption. The TAM framework informed the development of the topic list for interviews and guided the analysis by providing an interpretative lens to assess healthcare providers' experiences with the Afya Pro Connected Care app in managing diabetes and hypertension.(Appendix 2) By using TAM, we systematically analysed how providers perceived the app's usefulness and ease of use, as well as how these perceptions influenced their intentions and behaviors regarding app integration into routine practice (see Fig. 1).

## 2 Methods

This study involved two healthcare facilities in Ghana: Greater Accra and Eastern regions (Fig. 1). Two public outpatient facilities specializing in type 2 diabetes and hypertension management were purposefully selected to maximize variation in context and enable comparison across urban and semi-rural service environments, facility size and capacity, patient volumes, and accessibility. Purposeful (maximum-variation) selection was considered more appropriate than random selection, since the goal was to generate in-depth, contextually rich accounts of health-worker experiences with the Afya Pro Connected Care app rather than to provide nationally representative estimates.

The first clinic, Weija-Gbawe Hospital, is situated in the South-western part of Accra, formerly known as Ga South Municipal Hospital or “Akawe Hospital.” It employs 576 health workers, including 380 clinicians and 196 non-clinicians. Within the Weija-Gbawe Municipality, the hospital sees a daily admission rate of approximately 60 to 100 patients, with over 300 patients attending the outpatient departments (OPDs) and managing around 100–150 diabetes and hypertension patients weekly. The second clinic, Kwahu Government Hospital, is located in the semi-rural Kwahu South district of the Eastern region of Ghana. This district-run academic hospital serves an estimated population of 230,000 people from over 200 communities in the Kwahu municipal and beyond. The hospital offers diabetes care and general healthcare, boasting a substantial diabetes clinic that attends around 200 predominantly type 2 diabetic and 300 hypertensive patients weekly.

These geographically dispersed clinics range from approximately 7.2 km to 151.2 km from each other. By including facilities from different geographic, demographic, and organizational contexts, the study was able to capture diverse experiences of mHealth adoption across typical public-sector service delivery environments.

### 2.1 Study population

Both Weija-Gbawe and Kwahu Government Hospitals are secondary-level facilities providing diabetes and hypertension care. The study population comprised healthcare workers directly involved in chronic disease management, including doctors, nurses, physician assistants, and other relevant staff. Eligible participants had at least three months' experience using the Afya Pro app. Purposive sampling ensured maximum variation across professional roles, experience, and responsibilities to capture diverse perspectives on app use, rather than achieve national representativeness.

### 2.2 Recruitment and participation

Healthcare workers who had used the Afya Pro Connected Care app for at least three months were invited to participate in the study. This threshold ensured participants had sufficient hands-on experience to provide informed insights into the app's usability, integration into workflows, and impact on patient care. Invitations were extended via direct communication at the selected healthcare facilities. After obtaining informed consent, participants were assured of anonymity and confidentiality throughout the interview process. Recruitment was designed to include a diverse group of participants, ensuring variability in age, gender, professional roles, and work experience. All participants were proficient in English, and interviews were conducted in this language.

### 2.3 Scope of intervention and participants

The intervention was conducted in two health facilities across two regions, selected based on their established diabetes and hypertension management services. This targeted approach enabled a focused analysis of how the mobile health (mHealth) intervention impacted these specific clinical environments. Health workers directly involved in chronic disease management such as doctors, nurses, and allied healthcare providers were chosen as participants due to their hands-on experience and active engagement with the mobile app in patient care.

The study aimed to capture frontline healthcare providers' experiences at the primary care level, where much chronic disease management occurs. Although the pilot was not national in scope, the selected facilities provided valuable insights into the intervention's potential within typical healthcare settings for diabetes and hypertension care. By involving primary care staff managing these patients, the study explored the app's usability, benefits, and challenges in real-world practice, offering a practical perspective on integrating mHealth technology into routine care.

### 2.4 App usage in healthcare delivery

Healthcare providers accessed the app primarily via mobile phones for on-the-go convenience, using it two to three times daily. This frequency supported patient data monitoring, reminders, and consistent patient communication. Additionally, a secured hospital computer provided a secondary access point within a restricted common area, enabling authorized healthcare staff to conduct comprehensive data reviews and administrative tasks as needed. While the mobile app was the primary tool due to its portability, the hospital-based computer version served as a backup for more detailed data interactions.

### 2.5 Training and educational strategies

To ensure effective app use, healthcare providers received comprehensive training in three sessions of two hours each. These sessions were structured as interactive workshops combining hands-on practice, role-play scenarios, and demonstrations of key functionalities, including real-time monitoring and improved communication. Training materials included user manuals, video tutorials, and quick-reference guides to reinforce app usage skills. Training was organized at each facility's training room, enabling providers to work closely with facilitators and engage in peer learning. Following these sessions, participants were encouraged to share feedback and ask follow-up questions to address any operational challenges.

After providers had extensively used the app, in-depth interviews were conducted to capture detailed insights into their experiences, challenges, and the mHealth intervention's effectiveness. These interviews offered valuable perspectives on how the Afya Pro Connected Care app impacted diabetes and hypertension management within public healthcare facilities.

### 2.6 Data collection

In-depth interviews (IDIs) were conducted between January and March 2023 using a semi-structured interview guide informed by the TAM (Appendix 3). All interviews were conducted in English and took place within healthcare facilities, ensuring convenience and a comfortable environment for participants. Interviews were conducted during working hours to minimize travel burden and disruption to routine clinical activities, while acknowledging potential response bias. Each interview lasted 45–60 min, was audio-recorded, and transcribed verbatim, with field notes capturing non-verbal cues and contextual detail. Recruitment continued until thematic saturation was reached; no new themes emerged after 19 interviews, consistent with qualitative guidance that small, focused, information-rich samples can provide sufficient analytic depth. Thematic saturation was defined as no new codes emerging after two successive interviews, consistent with qualitative research standards.

## 3 Summary of interview guide

Semi-structured interviews were conducted with healthcare providers at the intervention facilities to explore the use of the mHealth app for diabetes and hypertension management. The interviews covered three main areas:

**Perceived Usefulness and Efficacy:** Participants were asked about their satisfaction with the app, training received, clinical usefulness, communication with patients, confidence in using the app, recovery from errors, and observed changes in patient outcomes since using the app. Barriers to app use and strategies to overcome them were also explored.

**Quality of mHealth Services and Service Satisfaction:** Questions focused on ease of use, learning curve, interface usability, time requirements, comfort using the app in social settings, confidence in clinical care, and recommendations for improving facility services. Participants also described the specific services available to patients through the app.

**Factors Influencing Implementation:** This section addressed how mHealth affects general care quality, task delegation to less qualified staff or patients/caregivers, coordination with other services, and recommendations for successful implementation at a national level. A full copy of the interview guide is provided in (Appendix 3).

### 3.1 Data analysis

Data analysis followed Braun and Clarke's six-phase thematic analysis approach, combining deductive coding (based on TAM constructs such as perceived usefulness, ease of use, and behavioral intention) with inductive coding to capture emergent themes (such as workflow integration, technical barriers, organizational support).

To enhance analytic rigor, the first and second authors independently coded an initial subset of transcripts to develop a provisional codebook. Themes were then identified by grouping the initial codes under TAM's key constructs, while new themes emerged inductively from the data. The research team engaged in iterative discussions to review and refine the themes, ensuring that they accurately represented both the theoretical model and the participants' experiences. Discrepancies were resolved through discussion and consensus, and the codebook was iteratively refined. This process, along with the maintenance of memos and an audit trail, ensured transparency and intercoder reliability. Atlas.ti version 9 software was used to manage coding. Researcher reflexivity was also considered. The team included both clinicians and social science researchers; to mitigate potential bias, we engaged in reflexive debriefings and discussed alternative interpretations throughout the analysis. We acknowledge that the research team's clinical and academic backgrounds may have influenced the interpretation of participants' perspectives, potentially emphasizing clinical or workflow-related aspects. To minimize bias, we engaged in reflexive debriefings, explicitly bracketed preconceptions, and triangulated interpretations across team members with diverse disciplinary perspectives. These steps ensured that multiple viewpoints informed the coding and thematic analysis. The study also acknowledged potential power dynamics, as participants might have felt hesitant to share negative feedback given the interviewers' association with the pilot team, and mitigated this by emphasizing confidentiality, creating a neutral interview environment, and encouraging honest and open responses.

### 3.2 Rigor and reporting

The final thematic report integrated TAM-related and emergent themes. Throughout, the study adhered to COREQ guidelines to ensure transparency and methodological rigor (Appendix 4).

### 3.3 Ethical approval

The study received ethical approval from the Ghana Health Service Ethical Review Committee (GHS-ERC0: 004/08/20). Informed consent was obtained from all participants after explaining the research objectives, procedures, risks, benefits, confidentiality, and participant rights. Participants could ask questions and voluntarily decide to participate, and only those who provided consent were included. To ensure confidentiality, all identifying information was removed from transcripts and datasets. Data were anonymized using unique codes, and no personal identifiers were stored with the research data. Electronic data were stored on password-protected, encrypted servers accessible only to the research team, while hard copies were kept in locked cabinets. Risks of deductive disclosure were minimized by aggregating data during reporting, avoiding the use of potentially identifying quotes, and ensuring that findings were presented in a manner that protected the privacy of individuals and communities.

## 4 Findings

The study involved 20 health workers, primarily nurses (12 participants) with over 10 years of experience, from the Greater Accra and Eastern regions of Ghana. All participants had used the AfyaPro Connected Care App. Qualitative analysis identified 16 key themes related to the acceptability, adoption, and feasibility of the mHealth intervention (see Table 1 for participant details). These themes were analysed using the TAM. To better interpret these findings, the results are presented using the TAM framework. The themes were mapped onto the core TAM constructs Perceived Usefulness, Perceived Ease of Use, Intention to Use, and Usage Behaviour while also considering external barriers and facilitators that influenced adoption.

**Table 1:** Demographic data of participants (*n* = 20).

Variables	Frequency
Health care providers	
Nurses	12
Medical officers	2
Physician assistants	3

Facility managing director	1
Public health nurses	2

Sex	
Male	9
Female	11
Age group	
20–29	10
30–39	8
>40	2

Educational qualification of the respondent	
Diploma	10
Degree	8
Masters	2
Years of work experience	
2–9 years	10
>10 years	10

### 4.1 How do health workers relate to and experience mHealth in delivering diabetes and hypertension care services?

#### 4.1.1 Perceived usefulness (PU)

**Understanding how health workers perceive the app's usefulness is critical for scaling mHealth interventions, as it affects adoption, workflow integration, and patient care outcomes.**

**These findings align with the TAM, where perceived usefulness drives adoption. However, health workers emphasized that infrastructure challenges, such as unstable internet or electricity, could limit the app's adoption even when its usefulness was recognized, showing that perceived usefulness alone is insufficient for sustained usage.**

#### 4.1.2 Improved efficiency, usefulness, and satisfaction

Health workers reported that the app simplified daily routines, streamlined communication, and reduced paperwork. Benefits were most evident when staffing levels were adequate. Most participants (*n* = 15) said the app simplified their workflow and improved efficiency by speeding up data access, while also facilitating collaboration among team members and enhancing information flow. This suggests that mHealth can meaningfully reduce administrative burden and improve team coordination.

“Because moving from the folders system has really helped, the new app now makes work easy, reduces delays, and simplifies client communication, also providing quick access and efficient coordination.” – **HW 8, male, BSc nursing, assistant ward manager.**

#### 4.1.3 Access to specialist care and follow-up

Access to specialist services and structured follow-up is a key component of chronic disease management.

Half of the participants (*n* = 10) noted that the app improved access to specialist services, including dietitian consultations, and strengthened patient education. They also noted that the app supported follow-ups that were previously lacking, such as weekly phone calls to check on medication intake and appointment adherence.

“Patients are now scheduled to see a dietitian once every visit, and education about good management techniques is intensified.” – **HW 3, female, Diploma Nursing, junior staff.**

“We conduct weekly phone follow-ups with patients, which we didn't do before the app launch.” – **HW 4, female, Nursing Assistant.**

#### 4.1.4 Impact on chronic disease care

A significant number of health workers (*n* = 13) advocated for scaling up the app nationally, citing its potential to strengthen care for diabetes and hypertension. While optimism was high, some emphasized that sustainability would depend on addressing infrastructure challenges, such as connectivity and electricity. This highlights the importance of pairing technological interventions with reliable infrastructure to ensure long-term impact.

“I propose expanding the app usage to regional and national levels for managing diabetes and hypertension. This will benefit both healthcare workers and clients.” – **HW 3, female, Diploma** Nursing, junior staff.

“It has improved patient management, but without stable internet and power, we may struggle to use it consistently.” – **HW 7, male, BSc Nursing.**

Beyond perceived usefulness, ease of use was also a key factor influencing how quickly health workers integrated the app into their routines.

#### 4.1.5 Perceived ease of use (PEOU)

Perceived ease of use (PEOU) facilitated confidence and adoption, supporting TAM predictions. Yet, limitations in patient digital literacy and access to devices moderated how effectively the app could be used in patient care, demonstrating that ease of use alone does not guarantee consistent engagement. Ease of use is a key determinant of sustained engagement with mHealth tools, influencing both health worker confidence and patient follow-up.

#### 4.1.6 Ease of use

Nearly all participants (*n* = 19) highlighted the app's intuitive and simple organization, which made it easy to navigate and retrieve information.

“The app has simplified data management by tracking all our patient interactions and provided clear instructions. It also displays patient information and scheduled appointment dates upon opening.” – **HW 4, female, Nursing Assistant.**

#### 4.1.7 Training and confidence

Most participants emphasized that periodic refresher sessions enhanced their skills, accuracy, and confidence in using the app. Training included registering patients, monitoring vitals, and managing appointments, which reinforced routine practice and reduced errors. Some participants noted that periodic follow-up training was still needed, especially for staff who joined later. This shows that structured training supports both accuracy and sustained app adoption.

“The training taught us to register patients, monitor vitals, track measurements on the app, and manage appointments easily. Overall, we learned a lot.” – **HW 2, female, BSc Nursing, junior staff.**

#### 4.1.8 Affordability and patient access

A smaller number of participants (*n* = 3) raised concerns about affordability and patient access, particularly the cost of devices and internet connectivity. While less frequently mentioned, these concerns highlighted important equity considerations for wider adoption.

### 4.2 Facilitating and inhibiting factors related to the implementation and adoption of mHealth

#### 4.2.1 Usage Behaviour/ workflow integration

TAM suggests that perceived usefulness and perceived ease of use of the app influence usage behaviour. However, workflow integration was limited by hierarchical restrictions, such as junior staff being unable to prescribe, indicating structural constraints can moderate usage behaviour despite high perceived usefulness and ease of use. These findings suggest that usability and role flexibility are key to maximizing the impact of digital tools.

#### 4.2.2 Enhanced confidence and workflow integration

Several participants (*n* = 12) reported that the app's organized interface increased their assurance in performing tasks and streamlined daily workflows. The majority noted that initial hesitations were quickly resolved once they realized the app was easy to navigate. Still, some providers highlighted a preference for maintaining in-person consultations alongside digital tools, suggesting that hybrid care models may be most acceptable.

“The app is fu; everything is well organized and easy to navigate.” – **HW 6, male, BSc Physician Assistant.**

“Even though I still want to interact with patients more physically, I could still choose to do things via the app.” – **HW 8, male, BSc Nursing, Assistant Ward Manager.**

#### 4.2.3 Teamwork and task sharing

Over half of the health workers (*n* = 11) stated that the app promoted teamwork, facilitated efficient handovers, and improved problem-solving between senior and junior staff. However, restrictions on junior staff entering prescriptions limited its full integration into workflows, suggesting the need for structured decision-support tools.

“It enables quick error resolution and smooth handovers between shifts.” – **HW 9, male, BSc Nursing, Ward Manager.**

“Most junior staff are not permitted to enter prescriptions… out of concern they might make mistakes.” – **HW 11, female, BSc Nursing.**

Confidence gained through workflow integration reinforced intention to use the app consistently.

#### 4.2.4 Intention to use

Health workers' intention to use the app was strongly influenced by perceived usefulness and ease of use. Yet, patient literacy, access to smartphones, and equity considerations moderated this intention, showing that behavioral intention is context-dependent in resource-limited settings. Health worker perceptions of the app's usefulness and ease of use influenced intention to use, supporting patient engagement and continuity of care.

Most participants (*n* = 13) indicated that patients monitored their blood pressure and sugar levels using the app, which staff believed enhanced adherence and enabled prompt interventions. Yet, the degree of empowerment was uneven, often depending on patients' literacy and access to devices.

“Patients are now actively using the app to enhance self-management, uploading their results for follow-up. Those without devices go to pharmacies for tests and input results into the app. Remote monitoring enables quick intervention.” – **HW 2, female, BSc Nursing, Diabetes Clinic In-Charge.**

#### 4.2.5 Follow-up and accountability

Half of the health workers (*n* = 10) reported that the app reinforced follow-up through calls and reminders, improving continuity of care, which improved continuity of care for patients traveling long distances. Consistent supervisory oversight was noted as important for sustained use.

“Regular follow-ups ensure progress monitoring; longer intervals previously led to lapses in care.” – **HW 3, female, Diploma Nursing.**

“Now the app allows easy reminders for patients about their treatment regimens.” – **HW 2, female, BSc Nursing, Diabetes Clinic In-Charge.**

#### 4.2.6 Affordability and service accessibility

A smaller group (*n* = 4) described how the app indirectly lowered costs for patients by reducing fees for services like dietician and physiotherapy consultations, which increased clinic attendance. However, inequities remain, as not all patients can afford smartphones or reliable internet.

“Since the app's launch, service fees have decreased, resulting in increased patient attendance.” – **HW 2, female, BSc Nursing, Diabetes Clinic In-Charge.**

“Some patients complain about not having smartphones or enough data to use the app effectively.” – **HW 7, male, Diploma Nursing.**

#### 4.2.7 External barriers

Understanding barriers is critical to sustaining mHealth adoption. Health workers viewed the app as a tool that streamlined routine tasks but occasionally increased workload due to time-consuming data entry and limited staffing.

Some health workers (*n* = 6) reported that the app occasionally increased their workload, particularly when data entry was time-consuming or required extra effort due to staff shortages. Other barriers included poor internet connectivity, power outages, and limited training. Participants described these issues as intermittent rather than constant, but highlighted infrastructure challenges that may limit scalability.

These external factors unstable electricity, internet disruptions, limited patient access to devices, and staff shortages extend TAM by acting as contextual moderators that can suppress actual usage even when perceived usefulness and perceived ease of use are high.

“It can be difficult to use the app at times, especially if you are the only nurse on duty and have to multitask to enter a lot of data. If you have to enter the same data for several patients while caring for them, it could be a time waster.” – **HW 11, female, BSc nursing.**

#### 4.2.8 Power and internet connectivity

Nearly half of participants (*n* = 8) identified unstable electricity and poor internet connectivity as the most significant barriers to app use. Outages often delayed service delivery and forced staff to temporarily suspend app-based tasks.

“Our biggest challenge is the lighting system. When there's a power outage, our non-automatic generator requires electricians to manually switch it on, causing delays before the system can be used again.” **– HW 12, female, Nursing Assistant.**

Several participants also noted that internet disruptions occasionally interrupted data entry, though when power and connectivity were stable, the app still reduced administrative burden compared to paper records.

#### 4.2.9 Patients' smartphone access and digital literacy

A smaller number of participants (*n* = 5) pointed to patients' limited smartphone access, costly internet data, and low digital literacy, particularly among elderly patients as barriers to equitable app use. These issues occasionally forced a reliance on paper-based records, undermining continuity of care. Those with access were more engaged in managing their conditions.

“Some elderly clients face challenges using the app due to technology difficulties and a lack of smartphones. They resort to traditional paper methods to record data, causing tracking challenges.” – **HW 2, female, BSc Nursing, Diabetes Clinic In-Charge.**

“Those who can afford smartphones now send their results directly on the app. It helps us follow up more quickly than before.” – **HW 6, male, BSc Physician Assistant.**

### 4.3 Limitations for junior staff

More than half of participants (*n* = 9) raised concerns about the limited role of junior staff in the app's use, particularly regarding prescribing or adjusting medications. While safeguards against errors are important, restricted functionality reduced efficiency in emergencies, suggesting the potential benefit of decision-support tools.

“Junior staff, like me, can't prescribe medication, especially in emergencies. Doctors use the app exclusively, while we oversee patients awaiting review. The app needs to offer a user-friendly option for us to be more involved.” – **HW 9, male, BSc Nursing, Ward Manager.**

“If the app had a guidance system, even junior staff could help with prescriptions under supervision. That would really ease the pressure on senior staff.” – **HW 4, female, Nursing Assistant.**

## 5 Discussion and conclusion

### 5.1 Discussion

This study demonstrates the potential of an interactive mHealth app to strengthen healthcare delivery, reduce administrative burdens, and improve patient outcomes in diabetes and hypertension care. Health workers reported greater efficiency, improved communication, and overall satisfaction, consistent with previous findings by Adotey-Delove et al. (2023) and Odendaal et al. (2020), which highlighted the role of mHealth in enhancing workflow efficiency [18,19]. However, as Iyanna et al. (2022), note, the introduction of new technologies can also disrupt workflows and generate resistance, underscoring that successful adoption depends on context, training, and careful implementation [20]. Task shifting has the potential to enhance teamwork in healthcare, but its success requires careful planning and robust support.

The user-friendly nature of the app was consistently highlighted as a key strength, supporting prior evidence that usability is critical for technology integration [21]. In contrast, research by Galavi et al. (2024) and Giebel et al. (2024) shows that complex interfaces can pose barriers to adoption [22,23]. Designing adaptable platforms that accommodate diverse user skills and preferences will therefore be essential for broader implementation.

In line with the TAM, the findings show that perceived usefulness and ease of use shaped health workers' acceptance of the mHealth app. However, contextual factors such as infrastructure, organizational support, and digital literacy also influenced these perceptions, suggesting that TAM could be refined to better reflect technology adoption dynamics in low-resource health systems.

Building on this, our findings support an adapted TAM for LMICs in which structural readiness and infrastructural dependability act as external moderators, shaping the relationships between perceived usefulness, perceived ease of use, and mHealth adoption. In LMIC settings, the effect of perceived usefulness and perceived ease of use on mHealth adoption is moderated by structural readiness and infrastructural dependability.

This context-aware perspective aligns with evidence suggesting that hybrid care models are most effective when digital tools complement rather than replace in-person consultations and are supported by clear workflows, provider training, and ongoing supervision. These models work best for patients with more complex needs or lower digital literacy, as embedding digital tools into routine care improves provider acceptance and continuity of care.

Beyond efficiency, the app fostered patient empowerment through improved follow-up, education, and communication. Training health workers increased their confidence and ability to support patients, aligning with evidence that patient engagement enhances adherence and outcomes [24]. More active patient participation, facilitated by the interactive mHealth app, may improve patient care outcomes [25].

Our study identified barriers to mHealth app implementation, such as power and internet issues, technical challenges for older users, and device limitations. Nonetheless, persistent barriers such as poor connectivity, unstable power supply, and limited smartphone access reflect systemic infrastructural challenges ([26,27]. Additionally, junior staff highlighted the need for decision support features to bridge skill gaps, a recommendation consistent with Odendaal et al. (2020) [19]. While such tools could enhance teamwork and task shifting, Siala & Wang (2022) caution against over-reliance, emphasizing the importance of combining digital support with critical thinking and robust training [28].This finding is consistent with Kesse Tachi et al. (2014), who discuss the persistent challenges of technology adoption in low-resource settings and emphasize infrastructure limitations as an ongoing struggle in healthcare technology implementation [29].

In our findings, both health worker characteristics and patient-level factors influenced adaptation to the mHealth app. Younger and more digitally literate staff reported greater ease of use, while older or less digitally experienced staff sometimes faced challenges. At the patient level, disparities in age, socioeconomic status, and gendered access to smartphones affected engagement and occasionally required continued reliance on paper records. These considerations are important for national rollout strategies to ensure equitable adoption and optimize the benefits of mHealth.

The suggestion to add decision support features for junior healthcare workers aligns with recommendations from Odendaal et al. (2020), who advocate for tailoring mHealth interventions to the specific needs and roles of healthcare workers [24].

Overall, these findings suggest that while mHealth apps hold considerable promise for improving chronic disease management in resource-limited settings, their long-term effectiveness depend not only on clinical outcomes but also on addressing infrastructural gaps, ensuring usability, and tailoring tools to the roles and needs of different health worker groups.

The study indicates generally positive perceptions of mHealth tools, participants also reported workload pressures and system delays as barriers to effective implementation. To enhance sustainability and usability, future mHealth interventions should be co-designed with frontline health workers to align with existing clinical routines, reduce duplication of tasks, and streamline documentation. Furthermore, integration with national health information systems and the provision of supportive training can help ensure that mHealth use complements rather than adds to existing workloads.

While this study focused on provider perspectives, findings from our related patient-focused research (Aovare et al., 2025) revealed comparable themes, including perceived benefits of mHealth for communication and care coordination, alongside concerns about digital literacy and infrastructure [17]. This convergence strengthens the overall interpretation of mHealth acceptability and highlights areas for system-wide improvement.

We acknowledge that findings are contextually bound to the two facilities studied and are not for intended broad generalization, however, purposeful maximum-variation sampling and saturation enhance transferability to comparable primary care settings. Participants may have provided socially desirable responses due to the perceived link between the research team and the intervention. Additionally, differences between the urban tertiary and semi-rural district hospitals may limit transferability to other settings with different infrastructure, patient populations, or resource availability.

Ghana's approach to mHealth, with targeted training, supervisory support, and alignment with national health policies, contrasts with experiences in countries such as Tanzania and Nigeria, where fragmented policy frameworks and variable infrastructure have limited scale-up. In Rwanda, centralized mHealth strategies and comprehensive training programs have facilitated broader adoption despite similar resource constraints. These comparisons reinforce that while Ghana demonstrates several enabling factors for successful implementation, contextual adaptation remains essential when applying lessons to other African settings.

A comparison with mHealth initiatives in other LMICs reveals notable contextual differences. For example, while Tanzania and Nigeria have struggled with fragmented policies and limited infrastructure, Rwanda has seen wider uptake driven by centralized digital governance and robust, system-wide training efforts. Ghana's experience, with targeted training, supervisory support, and policy alignment, underscores the importance of adapting interventions to local contexts and health worker needs. Implementation and sustainability considerations include integrating capacity-building in digital literacy, ensuring interoperability with national health information systems, strengthening supervision and peer support, and exploring cost-sharing or public–private partnerships to maintain long-term feasibility.

Beyond feasibility, the long-term success of mHealth interventions depends on sustainable infrastructure, ongoing training, and integration into existing health systems. Scalability requires reliable power and internet, interoperable systems, and careful financial planning to ensure equitable and lasting impact.

### 5.2 Innovation

This study makes an important contribution to understanding mHealth implementation in low-resource settings by foregrounding the perspectives of healthcare providers in Ghana. While many prior studies emphasize patient outcomes or theoretical feasibility, our research centers on the workforce directly engaging with digital tools, offering a pragmatic view of adoption and integration challenges in this specific context.

Rather than claiming broad novelty, the contribution of this study lies in the contextual depth it provides for Ghana, where infrastructural constraints, cultural considerations, and operational realities shape the everyday use of mHealth applications. By documenting how a health technology originally developed in other contexts was adapted to local realities, including inconsistent internet access, power outages, and variable digital literacy, this research illustrates the importance of tailoring mHealth tools to national health system conditions.

The study also identifies forward-looking strategies relevant for Ghana, such as integrating decision-support functions into provider workflows, developing role-specific digital literacy training, and promoting blended care models that combine digital and traditional service delivery. These insights move beyond technical adaptation to highlight systemic design considerations necessary for sustainable mHealth adoption.

In this way, the paper complements patient-focused evaluations by emphasizing the provider perspective and situating digital innovation within the lived realities of healthcare delivery in Ghana. This balanced framing shows how digital tools can be embedded into health worker workflows in ways that support more equitable and context-appropriate health system transformation.

### 5.3 Implementation implications

The findings of this study suggest several actionable steps for effective mHealth implementation and scale-up in Ghana and similar low-resource settings. First, continuous capacity-building for health workers, particularly in digital literacy and data management, should be embedded within national training programs led by the 10.13039/501100004726Ministry of Health (MoH) and supported by facility managers. Second, interoperability with existing national health information systems is essential to ensure data consistency, reduce duplication, and should be coordinated by mHealth vendors in collaboration with the MoH. Third, strengthening supervisory structures and peer support mechanisms can enhance sustained engagement and accountability; facility managers and team leads should oversee these activities while considering staff workloads to prevent overburdening healthcare providers. Finally, cost-sharing models and public–private partnerships should be explored to ensure financial sustainability and equitable access, particularly after donor funding ends. These steps, tailored to the roles of responsible stakeholders and mindful of long-term operational and financial constraints, can support the successful integration of mHealth innovations into routine primary care delivery.

Our findings also highlight that patient-level inequities including differences in gender, age, and socioeconomic status can influence the adoption and benefits of mHealth interventions. National mHealth policies must explicitly address these disparities to ensure equitable access and outcomes. To mitigate these inequities, policymakers should implement targeted strategies such as inclusive digital literacy programs, user-friendly app designs, and supportive training for underserved populations. Incorporating equity-focused monitoring mechanisms can further ensure that mHealth initiatives reduce, rather than exacerbate, disparities in care.

### 5.4 Conclusion

Health workers found the interactive mHealth app to be feasible and acceptable and point out the potential of mHealth applications to streamline healthcare workflows, enhance patient engagement and improve patient care. The findings support the broader adoption of mHealth interventions in the management of chronic conditions like diabetes and hypertension in Ghana. Addressing identified barriers is crucial for sustained success. To ensure sustained success, it is important that policymakers and health administrators address identified barriers by investing in infrastructure, integrating mHealth tools into existing health systems, and providing ongoing training and support for health workers.

While the findings are grounded in the Ghanaian context, several lessons may inform mHealth scale-up in other LMICs. Targeted training, reliable digital infrastructure, and structured supervision emerged as critical enablers of technology adoption and are likely generalizable to similar settings. Adaptation to local cultural attitudes, health system organization, and resource availability remains essential for successful implementation.

To support sustainable and equitable mHealth integration, policymakers should consider institutionalizing digital literacy programs within national health and nursing curricula, incentivizing telehealth adoption, and establishing mechanisms to monitor long-term patient outcomes. Future research should evaluate the impact of mHealth on clinical outcomes and health equity across diverse populations.

The study addressed health workers' experiences and implementation barriers of mHealth, offering insights to guide effective adoption and theory development in LMIC healthcare settings.

## CRediT authorship contribution statement

**Pearl Aovare:** Writing – review & editing, Writing – original draft, Validation, Software, Methodology, Investigation, Formal analysis, Data curation, Conceptualization. **Erik Beune:** Validation, Supervision, Resources, Methodology, Data curation, Conceptualization. **Felix P. Chilunga:** Validation, Supervision, Resources, Methodology, Data curation, Conceptualization. **Nicolas Moens:** Validation, Supervision, Resources, Methodology, Data curation, Conceptualization. **Eric P. Moll van Charante:** Validation, Supervision, Resources, Methodology, Data curation, Conceptualization. **Charles Agyemang:** Validation, Supervision, Resources, Methodology, Data curation, Conceptualization.

## Ethical approval

The study received ethical approval from the Ghana Health Service Ethical Review Committee (GHS-ERC004/08/20). Informed consent was obtained from all participants after explaining the research objectives, procedures, risks, benefits, confidentiality, and participant rights. Participants could ask questions and voluntarily decide to participate. Only those who consented were included in the study.

## Funding

Financial support from the Africa eHealth Foundation significantly facilitated various aspects of the study, including data collection and analysis.

## Declaration of competing interest

The authors declare no competing interests associated with the research, ensuring an unbiased presentation of the study findings without any potential conflicts of interest.

## Appendix A Supplementary data

Supplementary material 1 Image 1

Supplementary material 2 Image 2

Supplementary material 3 Image 3

Supplementary material 4 Image 4

Supplementary material 5 Image 5

Supplementary material 6 Image 6
