# Supplementary material for: The acceptability, adoption and feasibility of mobile health interventions for diabetes and hypertension care among Ghanaian healthcare workers
Source: PEC Innov. 2026 Jan 22;8:100456. doi: 10.1016/j.pecinn.2026.100456 (PMC12870867; doi:10.1016/j.pecinn.2026.100456)

***Appendix 1;* Background information about the interactive mHealth application (AfyaPro connected care)**

**Overview of the Connected Care App and Pilot Implementation**

The app (named: Afya Pro Connected Care app) was part of a pilot implementation. The pilot implementation of the app aimed to evaluate its effectiveness as a mobile health intervention for managing chronic diseases, specifically diabetes and hypertension. (https://appadvice.com/game/app/afyapro/1464879369) or https://ecareaccess.org/.

The Afya Pro Connected Care app is a versatile healthcare management platform designed to improve the management of chronic diseases, specifically diabetes and hypertension.(Appendix 1) The motivation behind the development of this app stems from the growing prevalence of diabetes and hypertension in many LMICs, where traditional healthcare delivery methods often fall short in providing timely and effective management of these conditions. According to the World Health Organization (WHO), the global burden of these diseases has led to significant health complications and mortality, necessitating innovative solutions that enhance patient engagement and clinical outcome(18)

The app was developed by a team of healthcare professionals and technology experts who recognized the need for a comprehensive tool that integrates various functionalities to address the multifaceted challenges faced by patients with chronic diseases. It includes features such as real-time patient monitoring, medication reminders, health education resources, appointment scheduling, and a secure communication platform, all aimed at empowering both patients and healthcare providers. (Appendix 1). Users can download the app from platforms like the Google Play Store or Apple App Store, which ensures accessibility for a broad audience.

Training was provided to healthcare workers and patients to facilitate effective app use. Health workers guided patients through the app’s functionalities, demonstrating how to track vital signs, schedule appointments, and access educational resources. This training was crucial in building confidence among users, enabling them to engage with the app effectively at home and maintain open lines of communication with their healthcare providers. Regular follow-up sessions were conducted to reinforce training and address any challenges users faced, enhancing overall engagement.

The pilot implementation provided valuable insights into the app's usability and its integration into existing healthcare systems. It was crucial to evaluate how the app interfaces with other programs within the health information system to ensure comprehensive chronic disease management. By collecting data on user interactions and health outcomes, the pilot aimed to identify best practices for scaling the app's use across a wider population.

Understanding the context of diabetes and hypertension management prior to the intervention was essential. Before the introduction of the Afya Pro Connected Care app, patients often faced barriers such as limited access to healthcare facilities, poor communication with providers, and a lack of educational resources. These challenges highlighted the necessity for an intervention that did not only provide remote monitoring but also fosters a collaborative relationship between patients and healthcare providers. By addressing these needs, the app seeks to improve health literacy and encourage self-management among patients, ultimately leading to better health outcomes.


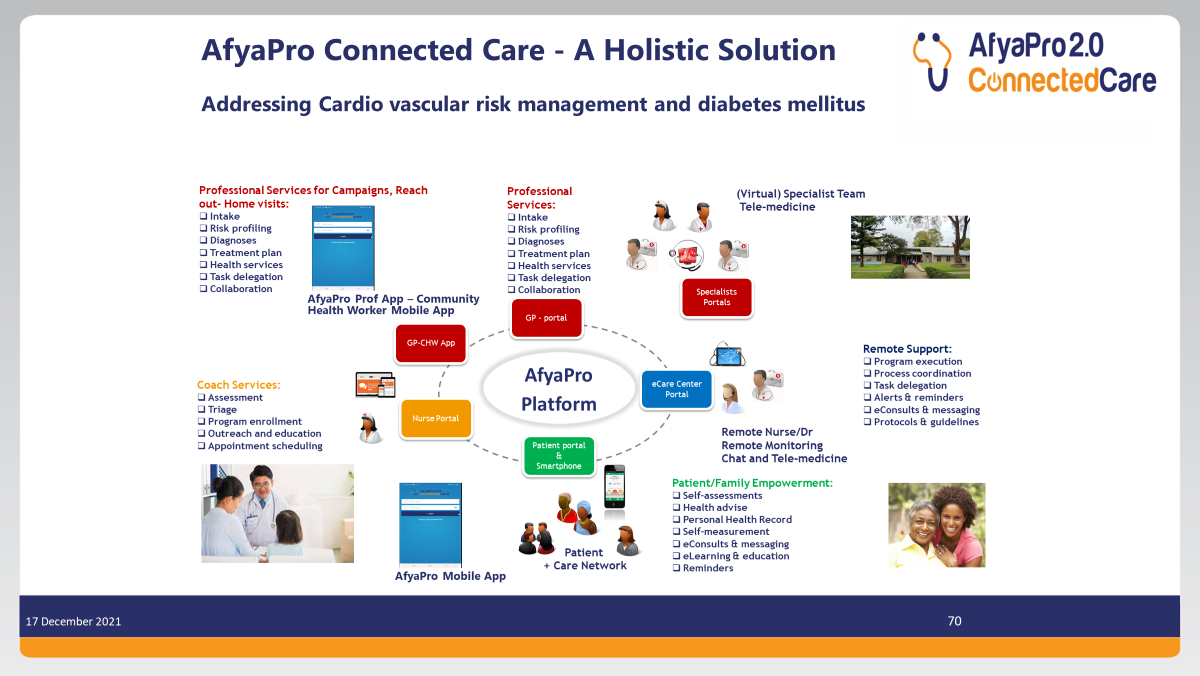


Summary of solution principles

**Implementation phases of the interactive mHealth application**

Step 1-Test the configured AfyaPro site and mobile App

Step 2-Assure all equipment is in place, networking done and connectivity is established

Step 3-The staff is trained on AfyaPro.

Two super users are identified and additionally trained.

Nearby IT support is defined and contracted to assist as needed.

Step 4-A plan for the migration of medical data is made

And put in motion. (Easiest is for the whole Clinic to migrate to AfyaPro)

Alternatively, experience can be obtained with AfyaPro for the CVRM-DM Clinic only.

A data clerk types over data as needed. As soon as the management of the Clinic is at ease with AfyaPro, the full migration can be done. Please note that integration is complex to maintain and expensive, could only be the case for a few selected EHRS).

Step 5-Final test of AfyaPro before going live

Step 6 -Enrol patients in AfyaPro. The enrolment of the patient in the CVRM-DM module is gradual. The nurse takes the vitals, patient history and does the risk scoring. Nurse indicates required lab test. Patient goes to lab for basic measurement Patient goes for doctor’s visit.

(Some additional lab tests might be needed with a revisit to the doctor)

Step 7-After the visit, the doctor re- introduces the patient to the nurse practitioner for education and a new appointment. Nurse might assign care tasks. (Doctor can bring patient physically to nurse or send a communication through AfyaPro). The nurse enrols the patient in the follow-up program that contains a care plan – and includes the use of the mobile App (by the patient or by family members).

Optional: To standardize further, the notion persona can be very effective (see Phase 1, step 11). Based on questionnaire a ‘persona’ is assigned to a patient. A persona describes the possible behaviour of the patient, and the care plan/ treatment can be adjusted accordingly. Based on the persona and the risk profile, the patient is introduced by the nurse to a follow-up program. Standard will be a follow-up consultation with the doctor every three months (to be decided by the doctor).

Note: In parallel arrangements can be doctor with nutritionists to complement the activities of the nurse. Even coaching and buddy groups can be considered that are moderated by the nurse or a coach. This should be defined during phase 1, step 9 or 10.

Step 8- Working session between doctor and nurse practitioner. To review patients as needed. For doctor to verify the work of the nurse practitioner (medical data, care plan, care tasks).For nurse practitioner to have her questions clarified.

Step 9 – Continuous activity – Dr to patient relation (patient centered), An important and continuous activity is the doctor showing his (digital) presence to the patient. The doctor- patient bond needs to remain strong. (This could be through the chat function in the mobile app)

Step 10 – Continuous monitoring. Measure the results (population management module in AfyaPro) and make the evidence clear, including feedback to the patients and their family.

Step 11- Evaluate and refine step 6 to 10 of phase 2 and improve accordingly to prepare phase Hand over officially, define follow up needs. (The AfyaPro-IDBH support will apply in phase 3 but is bound to maximum levels.

Note: During this phase the AfyaPro-IDBH contracted research staff will visit once or twice and conducts interviews to learn on satisfaction, points of improvement and impact.

**BACKGROUND READING SOME ILLUSTRATIONS OF AFYAPRO**

Below in figure 1, visually represents the versatile application of AfyaPro, showcasing its adaptability across various care programs and referral chain contexts. This illustration provides a clear understanding of how AfyaPro seamlessly integrates into different healthcare settings.

In Figure 2 below, a practical example demonstrates AfyaPro's role in supporting patient empowerment and remote care within an integrated proactive program. This visual representation offers insight into the platform's real-world implementation and its impact on patient care.

For a closer look at AfyaPro's user interface, Figure 3 below, presents a screenshot of the patient app. This glimpse into the interface provides a visual understanding of the platform's design and functionality. Additional screenshots can be explored in Annex 2 for a more comprehensive view.

**BACKGROUND READING SOME ILLUSTRATIONS** **OF AFYAPRO**


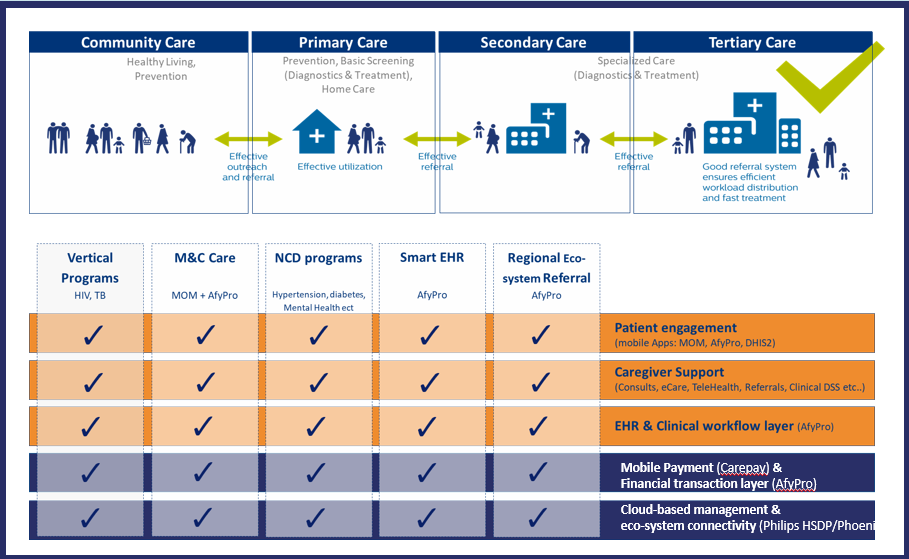


Figure1;Applicability of AfyaPro for different care programs


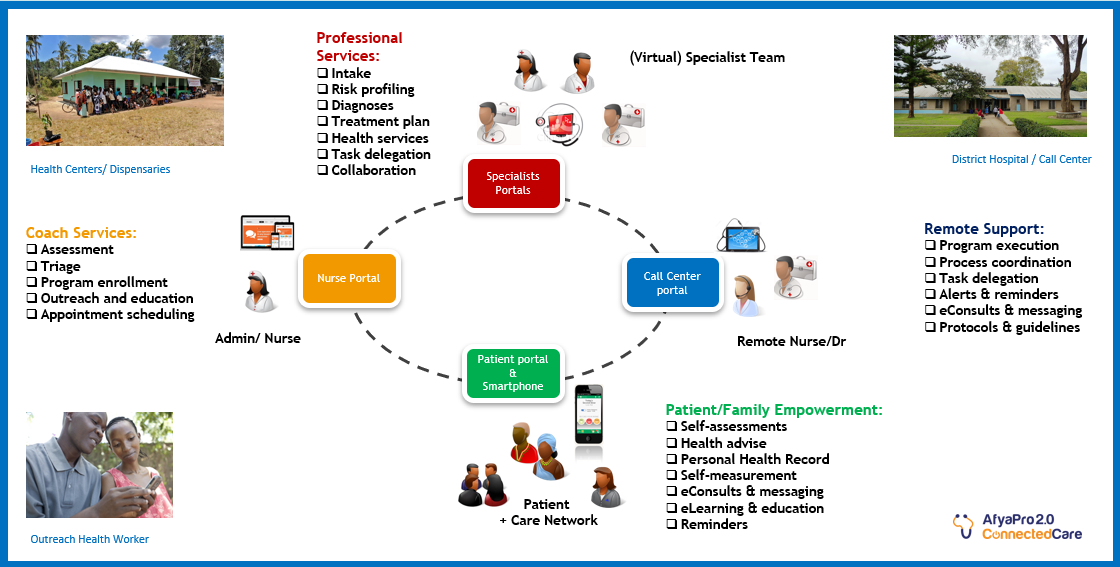


Figure2 AfyaPro used to support patient empowerment and remote care

Figure 3, Screenshot of the patient App


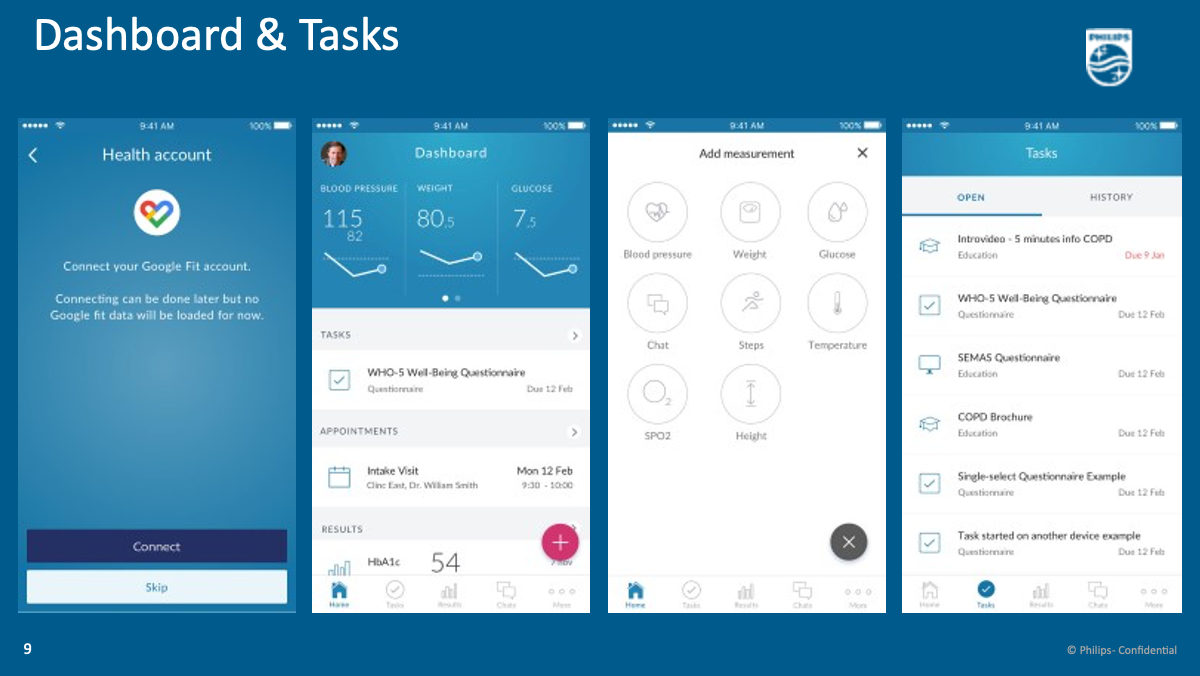


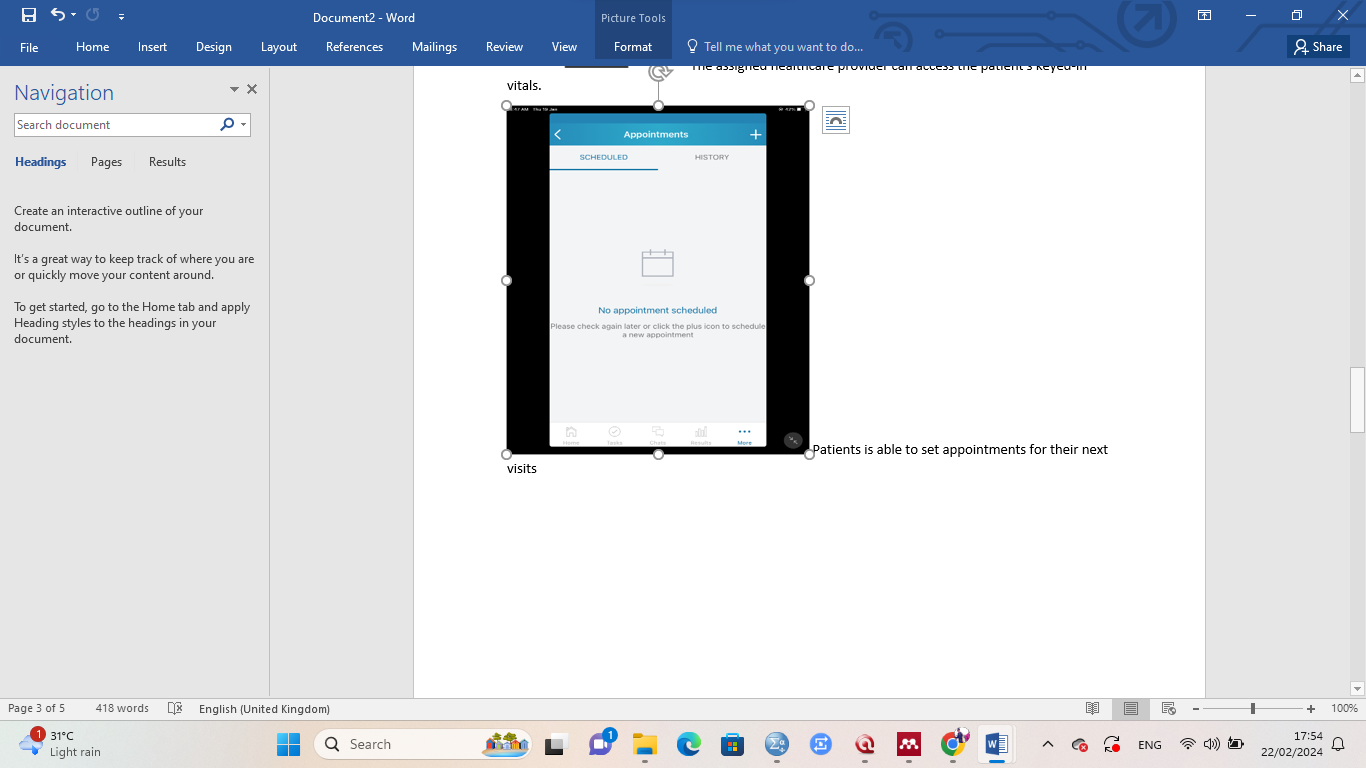

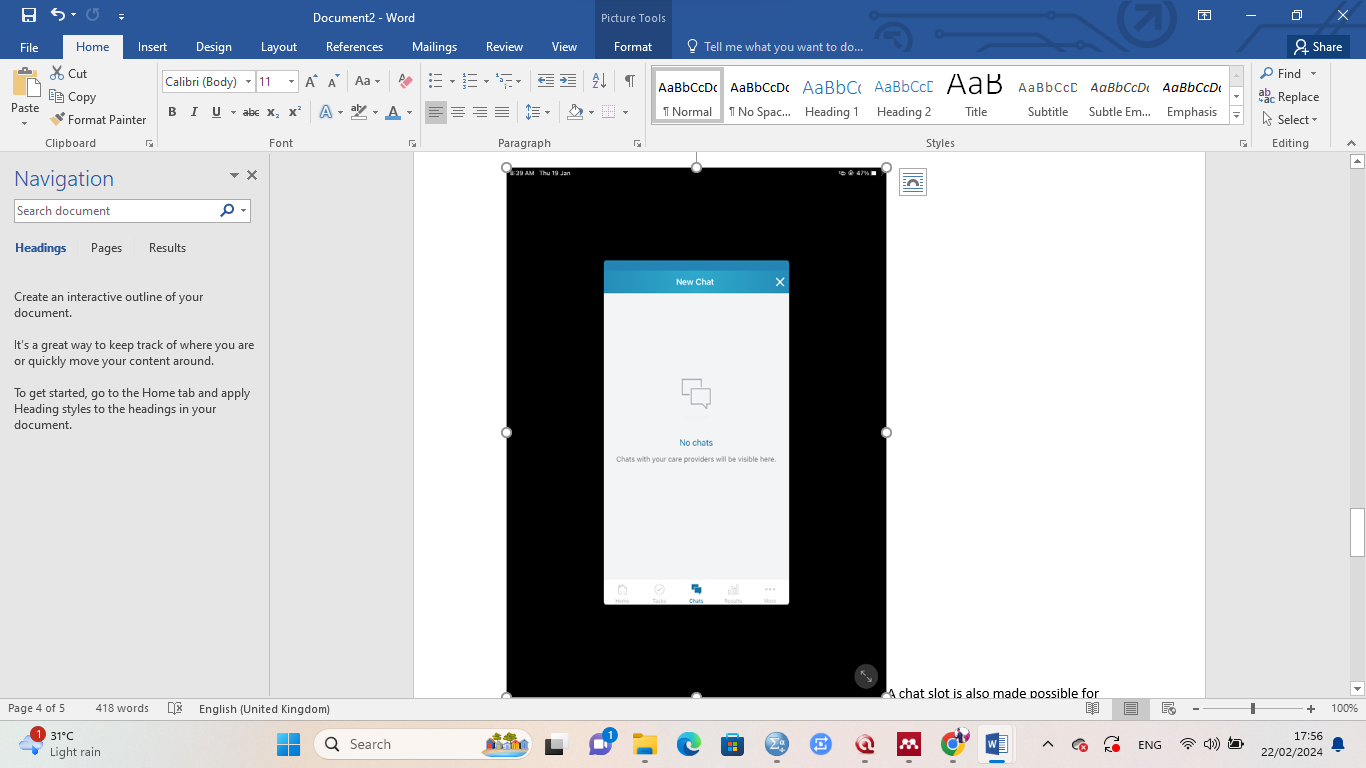

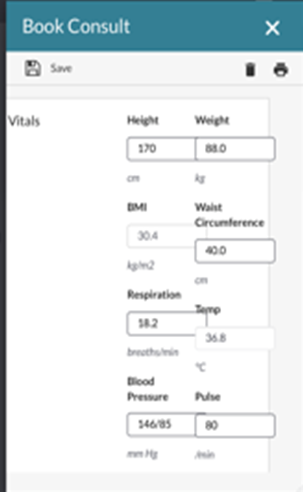


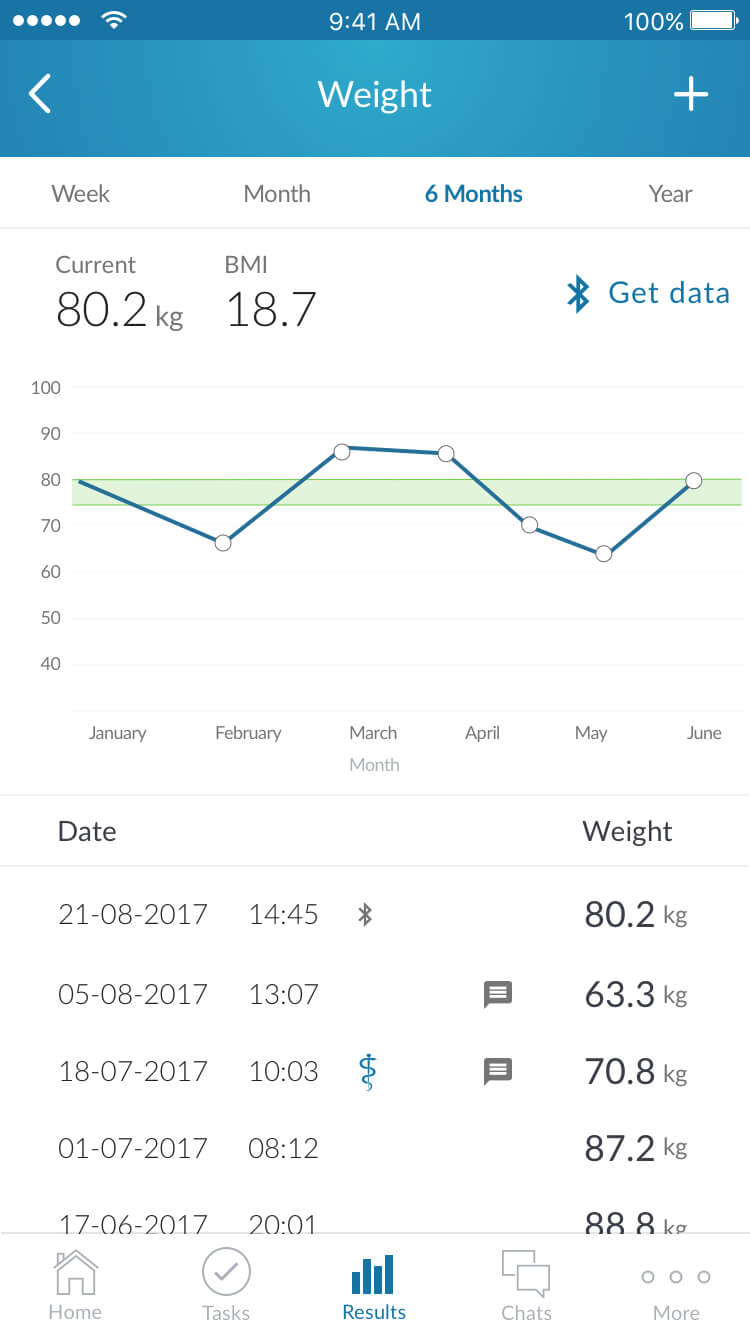

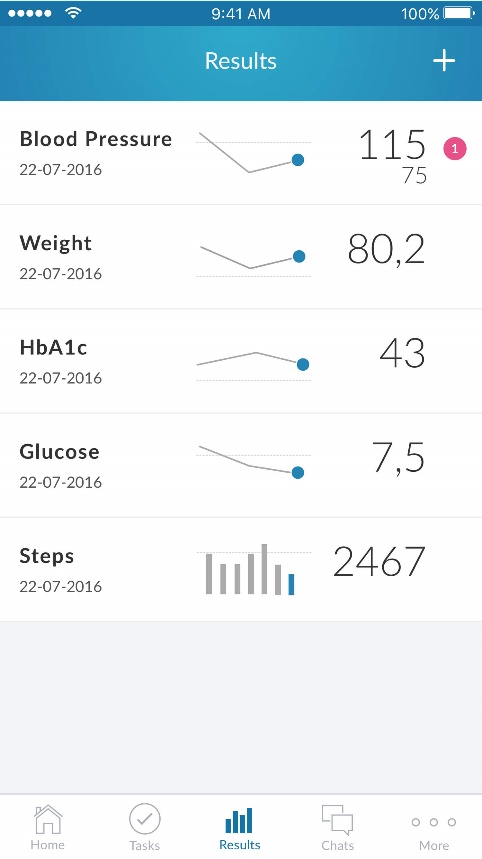


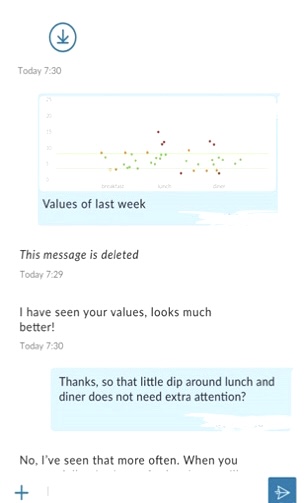


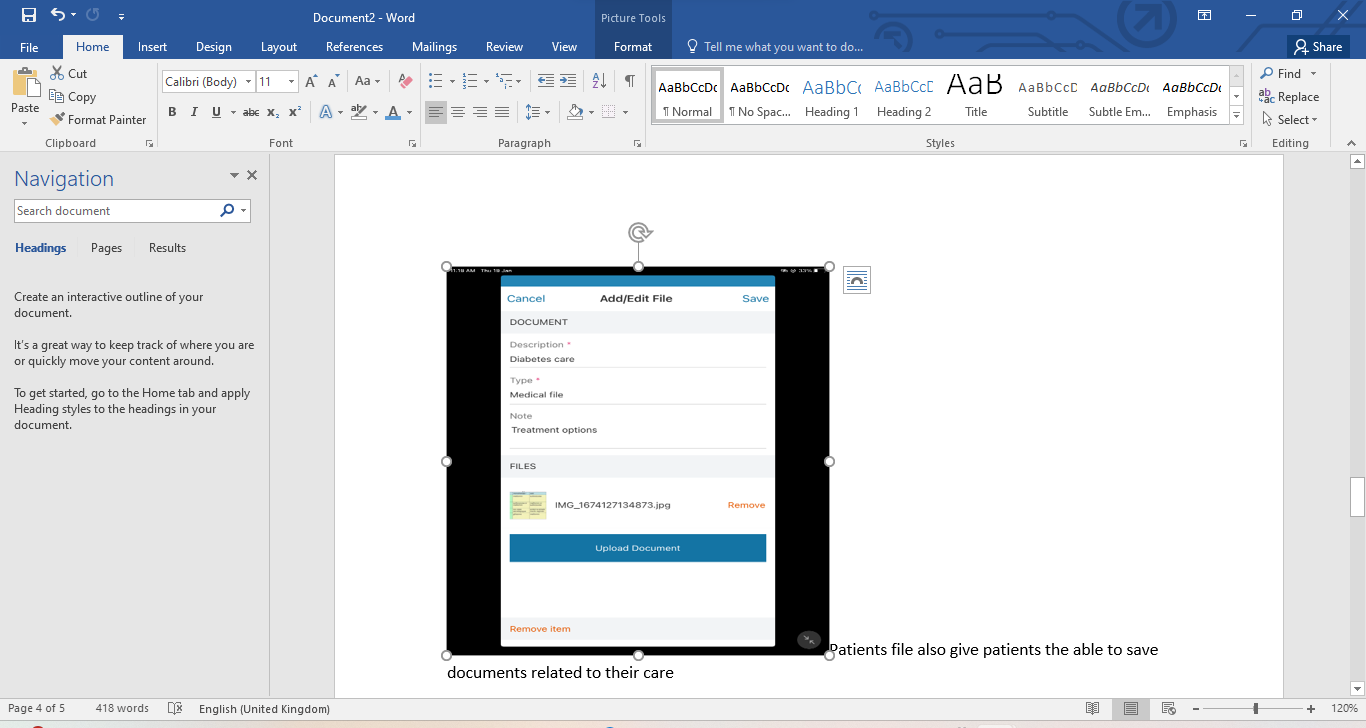

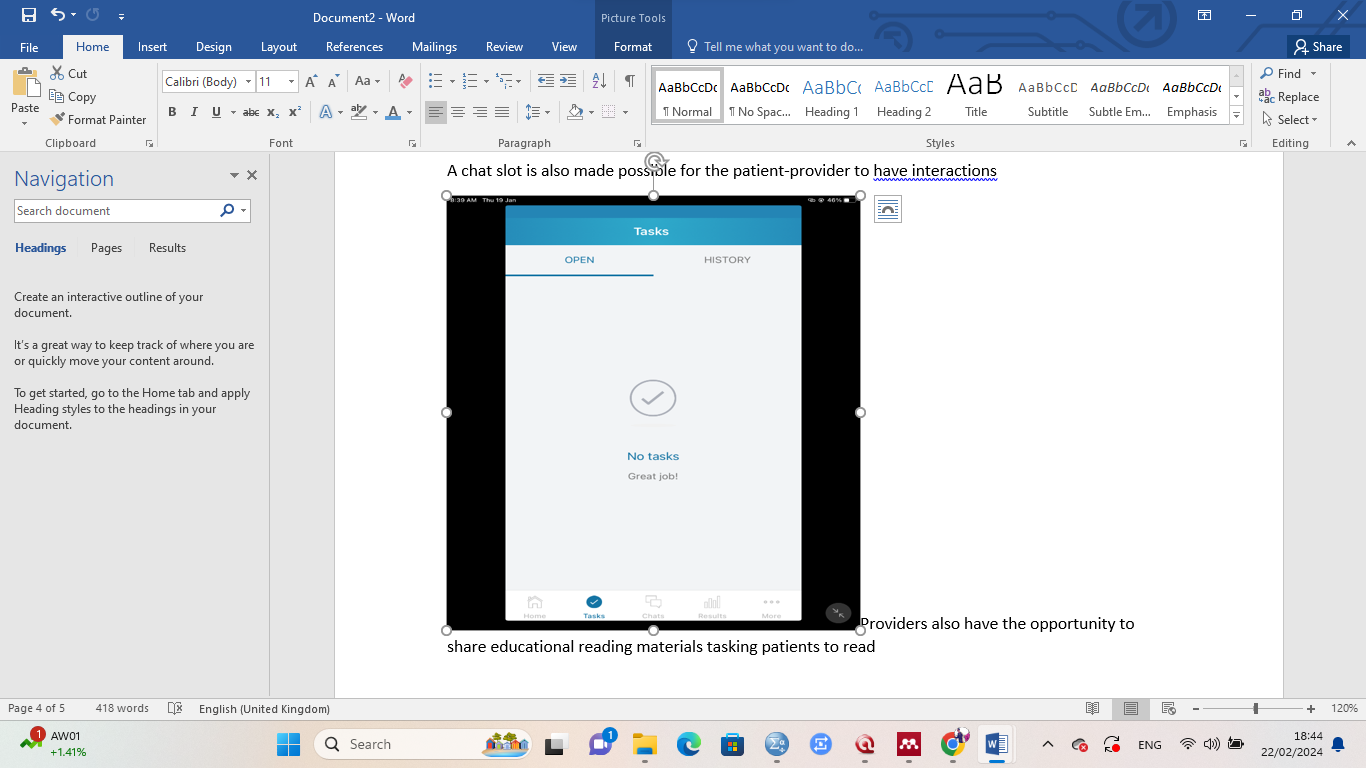

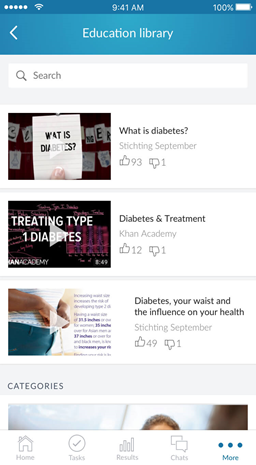

Supplement: Supplementary file 1 — Supplementary material 1 [file mmc1.docx]
