# Supplementary material for: The acceptability, adoption and feasibility of mobile health interventions for diabetes and hypertension care among Ghanaian healthcare workers
Source: PEC Innov. 2026 Jan 22;8:100456. doi: 10.1016/j.pecinn.2026.100456 (PMC12870867; doi:10.1016/j.pecinn.2026.100456)
Supplement: Supplementary file 2 — Supplementary material 2 [file mmc2.docx]

***Appendix 2;*** *Interview topic guide*

| ***Topic*** | ***Subtopic*** |
| --- | --- |
| *Researcher introduction* | Introduction interviewer, research group and sign informed consent |
| *Participants introduction* | *Demographics of health workers* |
| ***mHealth usefulness and efficacy*** | *-App satisfaction*  *-Training or education with the App*  *-Useful or important of the app in field of practice* |
| ***mHealth intervention on patients’ care*** | *-mHealth support for person with diabetes and hypertension*  *-clinical value of the app for patients with diabetes and hypertension*  *-Barriers to using mHealth in clinical practice*  *-App changing the delivery of health care* |
| ***Quality of mHealth services*** | *-App features and functions (easy to use,*  *easy to learn on, organized information on the app and the app interface)*  *-How comfortable using the app in social settings*  *-Amount of time involved in using the app*  *-How confident using the App to treat diabetes and hypertension patients*  *-Recommendations to improve care in the facility*  *-Special services are available by mHealth* |
| ***implementation of mHealth app*** | *-Special care services of mHealth affecting general care of diabetes and hypertension*  *-mHealth services affecting feasibility of delegating technical tasks to less qualified health workers*  *-Reflections on the process and challenges of mHealth services*  *- Recommendations for the implementation of a successful mHealth app* |
