# Supplementary material for: The acceptability, adoption and feasibility of mobile health interventions for diabetes and hypertension care among Ghanaian healthcare workers
Source: PEC Innov. 2026 Jan 22;8:100456. doi: 10.1016/j.pecinn.2026.100456 (PMC12870867; doi:10.1016/j.pecinn.2026.100456)
Supplement: Supplementary file 3 — Supplementary material 3 [file mmc3.docx]

***Appendix 3-Questionnaire***

***Introduction -Interviewer guide to assess the management of diabetes and hypertension patients***

My name is ______ I am from the university of Amsterdam. I am evaluating the ***effect of mHealth on the experiences and performance of health providers/facilities*** *to* help make changes that can improve quality of care for diabetes, hypertension patients and to also improve the work of the health workers in diabetes and hypertension management. We are here today to get input from the people who really work with the patients and the mhealth technologies in practice. We would like to ask you some questions about your experiences of using an mHealth to work in your diabetes/hypertension clinic or center. With this input, we can learn how to improve the support systems for Health care providers

We would like to conduct the interview in approximately 40 minutes and would be happy if we can audio record it. I hope this is possible. Your responses will remain confidential, and the information will not be linked to specific practice sites or individuals. Your participation in this study is voluntary. You may request at any time that we stop the interview or turn off the audio recording. Do you agree?

| Date of interview: | 1. DD | 1. MM | 1. YYYY |
| --- | --- | --- | --- |

| Respondent ID: | Phone No: | *(Intervention facilities) 1/2* |
| --- | --- | --- |

1. Facility: [1] St. Martins Hospital [2] Wenchi Hospital [3] Holy Family Hospital

1.**RESPONDENT’S BACKGROUND**

| 1. Age (in years): |
| --- |
| 1. Sex: [1] Female [2] Male |
| 1. Marital status: [0] Never married [2] Married [4] Widowed    - - 1. [1] Cohabiting [3] Divorced/Separated |
| 1. Religion: [0] None [1] Christian [2] Muslim [3] Traditional African    - 1. Other: |
| 1. Highest level of education completed……………………………………………………………… |
| 1. Current position/Unit…………………………………………………………………………………... |

***Section A: Perceived usefulness and efficacy of mHealth intervention***

*1.Overall, how satisfied are you with this app? Why?*

2. What training or education did you receive regarding the mHealth app in your facility?

3. How useful or important has the mHealth app been in your field of practice? ***Probe; How*** *does the app help you manage clients effectively?* ***Probe;*** *How convenient is the app for you to communicate with your clients?* what concerns do you have about using the mHealth app? **Probe***: How confident are you that any information you send to your clients using the app will be received?*

*4. How does the app allow you to recover easily or quickly when you make mistakes?*

*5. How does it provide an acceptable way to receive health care services?*

*6.How does the app adequately acknowledge and provide information for you to know the progress of your action?* ***Probe:*** *How does the interface of the app allow you to use all the functions (probe; entering information, responding to reminders, and viewing information) offered by the app?*

7. what are the most important elements of change since the moment you started using the mHealth app? **Probe:** a) what has changed/improved in the management of diabetes and hypertension?  *b)* *How well do patients control their blood sugar/blood pressure? c) What kind of information do patients have access to? Do they encounter challenges in managing their condition by using the app? How satisfied are your clients, if not why?*

8. How satisfied are you with the knowledge or concept of mHealth app being used in your facility? *Probe: What made you satisfied/dissatisfied about this app?*

9. What are the barriers in using the mHealth app in your field of work? **Probe:** how were barriers resolved?

**providers perception of mHealth intervention on patients’ care improvements**.

1.How can mHealth be used to support patient care among person with diabetes and hypertension?

2.What is the clinical value of the app for patients with diabetes and hypertension?

3.What are the barriers to using mHealth in clinical practice?

3.How is the app changing the delivery of health care among person with diabetes and hypertension?

**Section B: Quality of mHealth services and service Satisfaction (patient and facility perspective)**

*1. How easy Is the app to use? Probe how easy was the app to learn on?*

*2. How was information on the app organized?*

*3. What did you like about the interface of the app?*

*4. How comfortable do you feel in using the app in social settings?*

*5.What is the amount of time involved in using the app?* ***Probe:*** *is it fit for you?*

6. How do you feel *(confidence)* in treating diabetes and hypertension with the app?

***Probe*** *In caring for these patients what are some of your main challenges?*

7. What are some of your recommendations to improve care in the facility?

8. What special services are available by mHealth? Please describe the services available?

***(Probe-*** *for example, a person with diabetes or hypertension comes to this facility-then what would they need to do)?*

**Section C: factors influencing implementation of mHealth**

1. How does the special care services of mHealth affect the general care of diabetes and hypertension in your facility?

2. How does mHealth services affect the *possibility/feasibility of delegating some technical tasks to less qualified health workers and even patients/caregivers?*

3. What are some of your reflections on the process and challenges of mHealth services in your facility? ***(Probe-*** *where, how, do patients return, feedback from referred center, any coordination)*

*4. What recommendations will you suggest for the implementation of a successful mHealth app at national level?*
