# Supplementary material for: The acceptability, adoption and feasibility of mobile health interventions for diabetes and hypertension care among Ghanaian healthcare workers
Source: PEC Innov. 2026 Jan 22;8:100456. doi: 10.1016/j.pecinn.2026.100456 (PMC12870867; doi:10.1016/j.pecinn.2026.100456)
Supplement: Supplementary file 5 — Supplementary material 5 [file mmc5.docx]

# Coding Tree- Care Providers

| TAM Construct | Theme | Subtheme / Codes | Exemplar Quote |
| --- | --- | --- | --- |
| Perceived Usefulness | Improved Efficiency & Satisfaction | reduced paperwork; faster access; smoother communication; satisfaction | “The new app now makes work easy, reduces delays, and simplifies client communication.” – HW 8 |
| Perceived Usefulness | Patient Management & Monitoring | remote vitals entry; adherence tracking; monitoring improvements | “Patients now use it to enter their vitals… enabling better monitoring.” – HW 2 |
| Perceived Usefulness | Access to Specialist Care & Follow-up | dietitian referral; patient education; weekly phone calls; appointment adherence | “We conduct weekly phone follow-ups with patients, which we didn’t do before.” – HW 4 |
| Perceived Ease of Use | Ease of Use | user-friendly interface; simple navigation; quick retrieval of info | “The app has simplified data management by tracking all our patient interactions.” – HW 4 |
| Perceived Ease of Use | Training & Confidence | registration; monitoring vitals; managing appointments; need for refreshers | “The training taught us to register patients, monitor vitals, and manage appointments easily.” – HW 2 |
| Intention to Use | Patient Empowerment & Engagement | self-monitoring; uploading results; pharmacy test input; literacy differences | “Patients are now actively using the app… Remote monitoring enables quick intervention.” – HW 2 |
| Intention to Use | Follow-up & Accountability | calls; reminders; continuity of care; need for supervision | “Now the app allows easy reminders for patients about their treatment regimens.” – HW 2 |
| Usage Behaviour | Workflow Integration & Confidence | routine use; handovers; preference for hybrid care | “The app is user friendly; everything is well organized and easy to navigate.” – HW 6 |
| Usage Behaviour | Teamwork & Task Sharing | collaboration; error resolution; prescription restrictions | “It enables quick error resolution and smooth handovers between shifts.” – HW 9 |
| External Barriers | Connectivity & Power | unstable internet; outages; delays in service | “Sometimes the internet just cuts off… but when it works, entering data is far faster.” – HW 7 |
| External Barriers | Workload & Time Constraints | multitasking; heavy data entry burden | “It can be difficult… if you are the only nurse on duty and have to multitask.” – HW 11 |
| External Barriers | Patient Access & Digital Literacy | cost of devices; lack of smartphones; elderly literacy challenges | “Some elderly clients face challenges… resort to traditional paper methods.” – HW 2 |
| External Barriers | Limitations for Junior Staff | inability to prescribe; reliance on senior staff; decision-support suggestions | “Junior staff… can’t prescribe medication, especially in emergencies.” – HW 9 |
