# Supplementary material for: The acceptability, adoption and feasibility of mobile health interventions for diabetes and hypertension care among Ghanaian healthcare workers
Source: PEC Innov. 2026 Jan 22;8:100456. doi: 10.1016/j.pecinn.2026.100456 (PMC12870867; doi:10.1016/j.pecinn.2026.100456)
Supplement: Supplementary file 6 — Supplementary material 6 [file mmc6.docx]

**Key Facilitators and Barriers to mHealth Adoption**

| **Category** | **Key Facilitators** | **Key Barriers** |
| --- | --- | --- |
| Perceived Usefulness (PU) | - Improved workflow efficiency - Enhanced collaboration among staff - Access to specialist services and structured follow-up | - Infrastructure issues (unstable electricity, poor internet) |
| Perceived Ease of Use (PEOU) | - Intuitive, user-friendly interface - Comprehensive training and refresher sessions - Confidence in using the app | - Patient digital literacy and limited smartphone access |
| Workflow Integration | - Streamlined daily routines - Efficient handovers and teamwork - Reduced paperwork | - Hierarchical restrictions (junior staff unable to prescribe) - Limited staff to enter data |
| Patient Engagement | - Remote monitoring supports adherence - Facilitates patient self-management | - Unequal access due to cost of devices or internet data |
| Support & Training | -Regular training sessions and user support - Peer learning opportunities | -Need for ongoing refresher training for new staff |
| External/Contextual Factors | -Hospital-based computer as backup for data review - Support from facility managers | -Power outages, internet disruptions - Staff shortages increasing workload |
